# Idiopathic spontaneous compartment syndrome of the right lower limb: a case report

**DOI:** 10.1186/s13256-021-02683-y

**Published:** 2021-03-03

**Authors:** Sareesh Bandapaati, Rayno Navinan Mitrakrishnan

**Affiliations:** 1 Kettering General Hospital, NHS, Kettering, UK; 2 National Hospital of Sri Lanka, Colombo, Sri Lanka

**Keywords:** Compartment syndrome, Spontaneous, Acute

## Abstract

**Background:**

Acutely painful lower limb is a common presentation to the emergency department, and acute compartment syndrome is an important differential diagnosis to consider given the correct predisposing history and clinical presentation. However, idiopathic spontaneous compartment syndrome is an uncommon occurrence.

**Case presentation:**

A 54-year-old Caucasian man with no previous comorbidities presented with acute right-sided lower limb pain with classical symptoms showing gradual evolution. He had no other history of medical relevance and no preceding injury. Examination showed a marginally enlarged right lower limb with stretched skin and tenderness. Routine blood tests were normal including D-dimer levels. However, in the absence of any underlying risk factors, acute compartment syndrome was suspected on clinical merit and confirmed with magnetic resonance imaging. He underwent successful surgical intervention with fasciotomy and achieved good recovery.

**Discussion:**

Acute compartment syndrome, though commonly attributed to trauma, can occur due to varied causes. Spontaneous acute compartment syndrome is attributed to diabetes mellitus. Idiopathic acute spontaneous compartment syndrome occurs in the absence of either intrinsic or extrinsic risk factors and is rarely documented in the literature. This case highlights the importance of appreciating classical clinical signs and having the clinical acumen to consider an obvious diagnosis even in its rarer form of presentation.

## Background

Acute compartment syndrome occurs secondary to crush injuries or fractures resulting in increased interstitial pressure within a closed osteofascial compartment. This leads to impaired venous circulation, resulting in fluid extravasation and edema. The raised pressure causes impaired lymphatic drainage and subsequently affects arterial supply, causing tissue ischemia. The cascade of events gives rise to the symptoms due to increased pressure within the closed space. Acute compartment syndrome is a surgical emergency. The lower extremities are more often affected, and the territory can range from the buttock to the foot [1]. Acute compartment syndrome can also occur following prolonged mechanical immobility and positioning. Alternative causes include metabolic derangement, tumor and myositis of varying causes, including infections, drugs and alcohol, and medical or surgical intervention [2, 3]. In the presence of a known predisposing risk factor and a clear history, acute compartment syndrome can be suspected based on its classical clinical presentation of pain, pallor, paresthesia, poikilothermia and paralysis [1]. However, in the absence of any obvious risk factors, idiopathic spontaneous acute compartment syndrome is a rarely reported phenomenon and may pose a diagnostic challenge [4]. Delay in triage and diagnosis can have a lasting impact on patient morbidity and mortality, with medicolegal implications [5], highlighting the need for a low threshold of clinical suspicion and greater awareness of its presentation even in the absence of obvious etiology. In this report we present a case scenario of idiopathic spontaneous compartment syndrome.

## Case presentation

A 54-year-old Caucasian man with no known previous comorbidity developed sudden-onset severe right-sided calf pain. The pain occurred while he was taking a casual walk in the evening. The pain intensity gradually worsened over the next few hours. The patient also noted progressive swelling of the calf of the right leg, gradually extending to the ankle. He denied trauma and could not recall a history of animal or insect bite. There was also no recent history of immobility. He was on no medication, either prescription or over-the-counter. The symptom onset was spontaneous and unprecipitated. The patient sought initial pain relief and self-medicated with codeine-paracetamol 30/500 mg and ibuprofen 400 mg. Failure to achieve adequate pain relief, along with evolution of symptoms with the development of numbness on the same side below the knee, prompted the patient to seek medical assistance.

On clinical examination, the patient appeared to be in severe pain. He was afebrile with normal hemodynamic parameters, with a pulse rate of 85 beats per minute. Systolic and diastolic blood pressure measurements were 133 mmHg and 76 mmHg, respectively. Oxygen saturation was 97% on room air. His National Early Warning Score 2 (NEWS2) was 0 based on clinical assessment. Systemic clinical examination was normal. On examination of the lower limbs, the right calf measured 45 cm in circumference and the left calf measured 44 cm in circumference. On visual assessment there was no rubor or blisters. However, the skin appeared tense and stretched. The right leg and calf were tender to the touch. The right-sided dorsal and posterior tibial pulses were present and capillary refill time was < 2 seconds, which was normal. The anterior and peroneal compartment was visually tense, which was more evident with toe movement. A reduction in sensation to light touch was also noted.

**Fig. 1:**
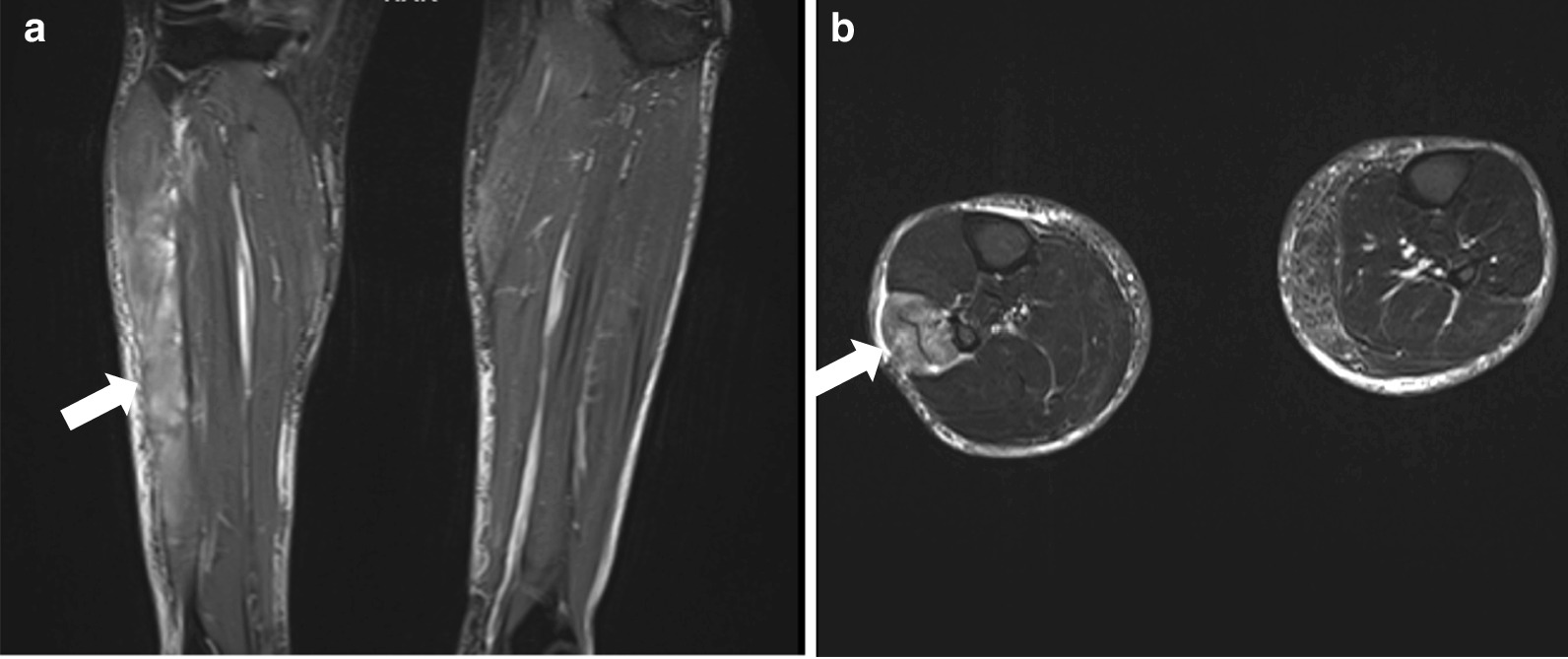
Contrast-enhanced magnetic resonance imaging of both lower legs: **a** coronal cut and **b** axial cuts. Both demonstrate (indicated by a white arrow) right-sided gross edema and swelling of the lateral lower leg compartment involving the peroneus brevis and longus muscles, consistent with acute lateral compartment syndrome. No intramuscular hemorrhage is evident. There is extensive soft tissue edema. Normal appearance of the anterior, deep and superficial compartments. Diagnosis favors acute lateral compartment syndrome of the right leg

Bedside venous blood gas analysis revealed a normal pH of 7.4 and a normal electrolyte profile, with blood glucose of 5.8 mmol/L (normal reference = 7–11.1). The serum lactate level was 1.6 mmol/L (normal < 1.8). Whole blood analysis revealed hemoglobin of 155 g/L (normal reference = 130–180), with a total white blood cell count of 9.9 × 10^9^/L (normal reference = 3.7–11) and a normal differential count. Platelet count was 275 × 10^9^/L (normal reference = 150–450). Renal and liver function and coagulation parameters were all normal. C-reactive protein was 4.3 mg/L (normal reference = < 5) and the D-dimer level was normal at 327 µg/L (normal reference = 0–500). Considering the clinical context and near normal biochemical parameters, the initial working clinical suspicion was compartment syndrome of the right leg despite the absence of an obvious etiology. This was based on the classical presentation, especially when D-dimers were negative, making deep vein thrombosis unlikely. Urgent magnetic resonance imaging of the lower limbs was done, which revealed gross edema and swelling of the right lateral leg compartment involving the peroneus and longus muscle, consistent with acute lateral compartment syndrome (Fig 1).

The patient received analgesia with intravenous administration of morphine 5 mg. The leg was kept in an elevated position. Urgent surgical consult was taken, and the patient underwent emergency anterior and peroneal compartment fasciotomy on the same evening of presentation. Subsequently he underwent a second-look washout and graft fasciotomy of the right lateral leg. He had an uncomplicated clinical course and underwent physiotherapy and recovered well, and was discharged 3 days after initial admission with a plan for review in 5 days to assess the graft and in 2 weeks to reassess the fasciotomy site. He was discharged on oral pain relief medication.

## Discussion and conclusion

An acutely painful lower limb is a common yet challenging presentation, with variable differential diagnosis. The usual causes include ruptured Baker’s cyst, deep vein thrombosis, cellulitis, muscular injury, tumor, arterial aneurysm, Achilles tendon rupture and acute compartment syndrome. A detailed history, clinical examination, basic blood workup and bedside ultrasound imaging usually helps narrow the differential diagnosis and clinch the correct diagnosis [6].

Acute compartment syndrome is usually suspected based on its classical presentation with the six P’s, which include pain, pulselessness and pallor, paresthesia and paralysis, and poikilothermia. These signs and symptoms manifest with rising intra-compartmental pressure (ICP) and are thus time-dependent. The diagnosis is mainly clinical, with ICP measurement being more of an adjunct and not a necessity. As not all the symptoms or signs may be present initially, it is important to have a low threshold for suspecting the diagnosis [2, 7]. Our patient presented the classical picture, thus aiding the clinician in early diagnosis despite the lack of an obvious insult or mechanism predisposing to this surgical emergency.

Spontaneous acute compartment syndrome is an uncommon phenomenon and is rarely documented in the literature. When described, spontaneous compartment syndrome is potentially associated with underlying diabetes mellitus (either type 1 or 2) in the context of poorly controlled glycemic levels [8]. The suggested hypotheses vary, and involve either diabetic muscle infarction from microangiopathic disease and subsequent edema secondary to infarction [9], or alternatively to osmotic fluid collection due to hyperglycemia and pressure-related ischemia, subsequently causing acute compartment syndrome [10]. However, our patient was not a diabetic. Furthermore, his blood sugar levels were within the normal reference range.

An uncommon cause for the development of acute compartment syndrome has been attributed to creatinine supplementation taken by athletes and individuals when engaging in intensive physical exercise [11]. Alternatively, acute exertional compartment syndrome has been observed in sedentary individuals who suddenly over-enthusiastically engage in exercise [4]. Our patient had no obvious risk factors, intrinsic or acquired, that predisposed him to developing acute compartment syndrome, making his situation an idiopathic spontaneous compartment syndrome. This is a very uncommon phenomenon, rarely reported in the medical literature, with only a few case reports to date [4, 12].

Acute compartment syndrome is an important surgical emergency which should not be missed. Classical presentation and clinical acumen should help in clinching the obvious diagnosis, even in its rarer forms of presentation.

## Data Availability

The datasets used and/or analyzed during the current study are available from the corresponding author on reasonable request.

## Abbreviation

ICP: Intra-compartmental pressure
